# The Impact of Different Telerehabilitation Methods on Peripheral Muscle Strength and Aerobic Capacity in COPD Patients: A Randomized Controlled Trial

**DOI:** 10.3390/arm92050035

**Published:** 2024-09-20

**Authors:** Amine Ataç, Esra Pehlivan, Fulya Senem Karaahmetoğlu, Zeynep Betül Özcan, Halit Çınarka, Mustafa Çörtük, Kürsad Nuri Baydili, Erdoğan Çetinkaya

**Affiliations:** 1 Department of Physiotherapy and Rehabilitation, Faculty of Health Sciences, Istanbul Gedik University, Istanbul 34876, Turkey; amineatac@gmail.com; 2 Department of Physiotherapy and Rehabilitation, Faculty of Hamidiye Health Sciences, University of Health Sciences, Istanbul 346668, Turkey; 3 Department of Physiotherapy and Rehabilitation, Hamidiye Institute of Health Sciences, University of Health Sciences, Istanbul 346668, Turkey; fulyakaraahmet@gmail.com (F.S.K.); zbetulozcan@gmail.com (Z.B.Ö.); 4 Department of Chest Disease, Yedikule Chest Diseases and Thoracic Surgery Training and Research Hospital, University of Health Sciences, Istanbul 346668, Turkey; halit.cinarka@sbu.edu.tr (H.Ç.); mustafa.cortuk@sbu.edu.tr (M.Ç.); ecetinkaya34@yahoo.com (E.Ç.); 5 Health Institutions Management Program, Hamidiye Vocational School of Health Services, University of Health Sciences, Istanbul 346668, Turkey; kursadnuri.baydili@sbu.edu.tr

**Keywords:** action observation, COPD, motor imagery, pulmonary rehabilitation, telerehabilitation

## Abstract

**Highlights:**

**What are the main findings?**

- Active muscle strengthening program has the same benefits as applying muscle strengthening program with action simulation methods to respiratory patients
- Telerehabilitation involving motor imagery and action observation is as effective as pulmonary telerehabilitation involving active strengthening exercises.

**What is the implication of the main finding?**

- Motor imagery and action observation methods may be alternatives to increase the effectiveness of a strengthening program and active exercise amotivation in COPD patients.
- Cognitive rehabilitation can be a powerful alternative rehabilitation method for respiratory patients who cannot tolerate active exercise programs.

**Abstract:**

Lung diseases have profound effects on the aging population. We aimed to hypothesize and investigate the effect of remote pulmonary telerehabilitation and motor imagery (MI) and action observation (AO) methods on the clinical status of elderly chronic obstructive pulmonary disease (COPD) patients. Twenty-six patients were randomly assigned to pulmonary telerehabilitation (PtR) or cognitive telerehabilitation (CtR) groups. The programs were carried out 3 days a week for 8 weeks. The 6-min walk test (6MWT), modified Medical Research Council dyspnea score, blood lactate level (BLL), measurement of peripheral muscle strength (PMS), and electromyography activation levels of accessory respiratory muscles were the main outcomes. There was a statistically significant improvement (*p* < 0.05) in both groups in the 6MWT distance and in secondary results, except for BLL. Generally, in the mean muscle activity obtained from the electromyography measurement after the program, there were statistically significant increases in the PtR group and decreases in the CtR group (*p* < 0.05). There was a statistically significant increase in PMS in both groups. An active muscle-strengthening program has the same benefits as applying the muscle-strengthening program to the patient as MI and AO. CtR can be a powerful alternative rehabilitation method in respiratory patients who cannot tolerate active exercise programs.

## 1. Introduction

Telerehabilitation involves using information and communication technologies to provide remote rehabilitation services to people in their homes [1]. It provides synchronous or asynchronous access to the rehabilitation environment for healthcare professionals and patients by telephone, video conference, and video recording. Two systematic reviews have investigated the costs associated with telerehabilitation. Kairy et al. [2] showed that lower costs for health services resulted from telerehabilitation. Del Pino et al. [3] found that telerehabilitation may be less costly and burdensome than in-person rehabilitation in a clinic for people with neurological and cardiovascular diseases. An important tool in the field of providing remote physiotherapy services is the technology of “TeleRehab”, which is short for telerehabilitation. TeleRehab is a form of telehealth that is accessible to anyone with just a mobile phone and reduces the burden of disease in the healthcare system by providing healthcare services such as continuous physiotherapy. Using a mobile application facilitates communication between healthcare personnel such as doctors and physiotherapists and the patient and minimizes costs [4].

Pulmonary rehabilitation (PR) is recognized as one of the most effective treatments available for elderly people with chronic obstructive pulmonary disease (COPD) [5]. PR promotes lifestyle changes and improves exercise capacity, quality of life (QOL), and dyspnea [6]. Recent studies comparing unsupervised home-based PR with standard programs, with or without the aid of digital technology, have shown promising results regarding common outcome measures [7]. 

Cognitive rehabilitation (CR) is a structured set of therapeutic activities. However, evidence for the effectiveness of CR is weak. The obvious effects of exercise, one of the main components of PR, are also linked to neurocognitive and neuroendocrine pathways [8]. Motor imagery (MI) is a treatment modality used in CR. Motor imagery, also known as kinesthetic imagery, is mental imagery that is more specific to motor context. Motor imagery is an active cognitive process whereby the representation of a particular action is internally reproduced (simulated) in the working memory without any explicit motor output. Importantly, researchers suggest that motor imagery provides a projection onto the action representation process by projecting an internal action representation [9]. Action observation (AO) involves the deliberate and structured observation of a movement [10]. In the last decade, AO has been proposed as a treatment modality in rehabilitation medicine in addition to traditional physiotherapy [11]. Since MI and AO act with mirror neurons in motor control and these neurons are interconnected in various cortex areas of the brain, these methods can cause the same plastic changes in the motor system when applied repeatedly [12]. Although MI- and AO-based interventions have been shown in the literature to contribute to improvements in neurology, sports, orthopedics, hand burns, tendon surgery, and musicianship, there is limited research in the literature on the effectiveness of their use in lung diseases, implemented as a comprehensive rehabilitation program. As far as is known, there are limited studies in the literature that integrate motor imagery and the action observation method with pulmonary rehabilitation and apply them to severe stage lung patients with the telerehabilitation method [10,11,13]. It is important to investigate methods such as MI and AO, which are methods that can be used during periods when active exercise is difficult, since peripheral and respiratory muscle dysfunction, and low quality of life [14,15], along with decreased movement of the patients, prolonged bed rest, and lack of motivation [16], may occur in elderly COPD patients.

It is acknowledged that the burden of COPD is expected to increase and is expected to become the third leading cause of death by 2030. Additionally, COPD has serious progressive adverse effects on daily symptoms, functional ability, and health status [17]. COPD, a preventable and treatable disease, is characterized by shortness of breath, cough and/or sputum production, and a permanent restriction in airflow due to abnormalities in the respiratory and alveolar pathways caused by particles or harmful gases [18].

Most studies of telerehabilitation in COPD are nonrandomized descriptive feasibility studies and have reported promising effects on symptoms, physical function, and quality of life [19]. We questioned how the effect of remote rehabilitation systems in the elderly population, who may have difficulty in performing active exercise and accessing rehabilitation services, would be, especially with the mental practical training of the elderly in addition to active exercise. In this context, we investigated the effect of telerehabilitation methods by adapting pulmonary telerehabilitation, which has been studied very little in the literature in the elderly population, to cognitive rehabilitation. The aim of this study is to investigate the effectiveness of comprehensive pulmonary telerehabilitation (PtR) and cognitive telerehabilitation (CtR) treatments using MI and AO, which are CR methods, in severe stage COPD patients who have difficulty in performing heavy exercise. 

## 2. Materials and Methods

### 2.1. The Study Design and Participants

This researcher-initiated, single-center, randomized controlled, and open-label study was conducted on severe COPD patients. This study was conducted in accordance with the Declaration of Helsinki, and approved by the Institutional Review Board (or Ethics Committee) of Yedikule Chest and Dıseases and Thorac Surgery Traınıng Research Hospıtal (protocol code 2021-131, 1 July 2021). The clinical trial record of this study is NCT05222295. Patient consent was obtained from all patients with a consent form. Inclusion criteria included age between 45 and 80 years, diagnosed with C and D group COPD according to GOLD staging, not using an assistive device, being on the same drugs for the last 4 weeks, receiving permission to participate in exercise from the responsible chest physician, achieving a score of 24 and above in the standardized mini mental state examination (MMDM), having no additional comorbid diseases of the orthopedic, neurological, and cardiac systems. The kinesthetic and visual imagery questionnaire-20 (KGIA-20) visual imagery and kinesthetic imagery score were determined to be 30 or more in both. Exclusion criteria of this study were the presence of a respiratory system disease other than COPD, presence of contraindication for moderate-intensity exercise, patients with acute exacerbation of COPD in the last four weeks, use of oral corticosteroid medication in the last four weeks, patient having a COPD exacerbation while research is ongoing, not understanding verbal instructions and being visually impaired, having participated in another clinical trial in the past thirty days that currently may affect the results of this study, and lack of technological equipment, e.g., internet or computer.

### 2.2. Randomization and Masking

**Figure 1:**
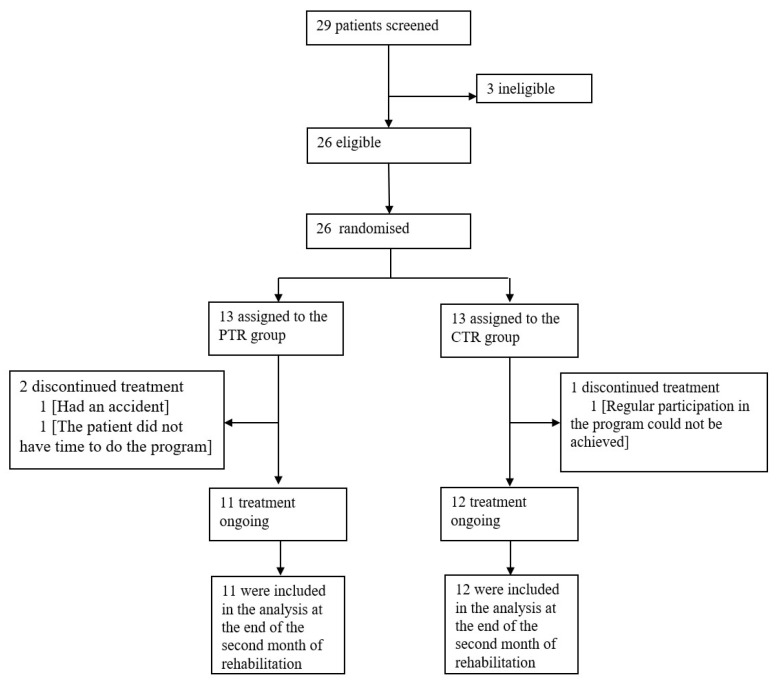
CONSORT flow diagram. COPD—chronic obstructive pulmonary disease; n—number of patients; PtR—pulmonary telerehabilitation; CtR—cognitive rehabilitation group.

Patients were randomly assigned to the synchronized supervised PtR group or CtR group in a 1:1 ratio using the randomization website (https://www.randomizer.org/) (accessed on 12 October 2021) [20]. The trial was open-label, so patients and researchers were aware of the treatment, but each patient was interviewed alone to mask different treatments, ensuring that they did not coincide with other patients included in the hospital evaluation in this study. The CONSORT participant flow diagram is presented in Figure 1.

### 2.3. Outcomes and Measures

**Figure 2:**
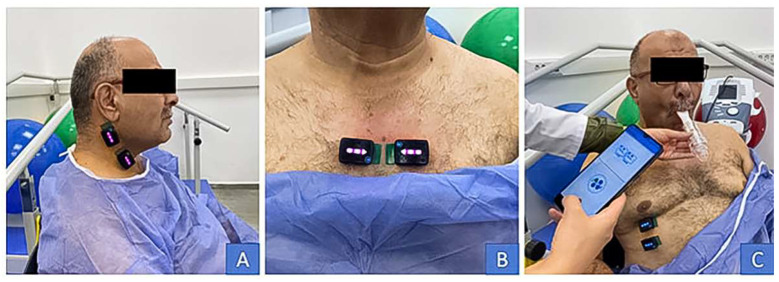
Placement points of the EMG sensors. (**A**) EMGscm; (**B**) EMGpara; (**C**) EMGdi.

The primary outcome measure was the six-minute walking test distance (6MWT) that assesses exercise capacity. As secondary outcome measures, an electronic hand dynamometer (Lafayette Instrument Company, Lafayette, IN, USA) was used to measure peripheral muscle strength. Patients’ dyspnea was evaluated with the mMRC scale, and blood lactate levels were evaluated with a portable lactate measuring device (Lactate Scout 4) after 6MWT. Activities of daily living were determined by the London chest activity of daily living (LCADL) scale [21], quality of life was determined by the Saint George Respiratory Questionnaire (SGRQ), and anxiety and depression were determined by the hospital anxiety and depression scale (HADS). Respiratory muscle activities (sternocleidomastoid (EMGscm), parasternal (EMGpara), and diaphragm (EMGdi) muscles) [22] were analyzed using surface electromyographic (EMG) measurement sensors (Delsys Trigno^®^ Avanti EMG Sensors, Delsys Inc., Natick, MA, USA) and EMG logger mobile application. The placement points of the EMG sensors are shown in Figure 2. EMG measurements were performed at rest with a two-minute break between them, while inspiring with an IMT device (Threshold IMT, Philips Respironics, Inc., Murrysville, PA, USA) at 7 cmH_2_O, 14 cmH_2_O, and 21 cmH_2_O inspiratory pressure [23].

### 2.4. Procedures

Before starting the rehabilitation, evaluations were made by the chest specialist doctor and the responsible physiotherapist before the telerehabilitation programs at the end of the 8th week in the hospital. For safe exercise, the patients were given a digital sphygmomanometer (Freely Bp-2208) and a digital finger pulse oximetry device (Yonker 81-C) by the researchers.

A group-based, supervised, and standardized PtR program was applied by the videoconference method at home three times a week for eight weeks. The exercise intensity ranged from three to five on the modified Borg scale and patients should be able to speak comfortably when speaking during the exercises. The tele-exercise program consisted of warm-up exercises, breathing exercises, active breathing techniques (ACBT), aerobic exercise (ground-based walking exercise), resistance training with THERABAND Trusted Progression™, and cool-down period.

For the CtR, MI + AO methods were applied. A video recording containing detailed verbal and subtitled commands, including MI and AO methods, each taken at anterior-lateral camera angles to match the number of exercise repetitions, was sent to the patients by the physiotherapist. Patients were asked to watch each exercise in the videos and then visualize them in their minds, following the instructions in the video while seated, with an eye–screen distance of sixty cm [24]. While watching each exercise, the instruction to imagine and try to feel the possible real emotional by following the commands given as if they were actually doing the exercise was included as a command in the videos. During the first five seconds of each exercise video, the participant’s position and which exercise they were following were expressed in writing and audibly. During the AO method, each exercise was replayed in the video for ninety seconds and a break was made between each exercise for thirty seconds. In the MI method, patients were asked to imagine each exercise as if it were real for forty-five seconds and then to rest for fifteen seconds as per the commands in the videos [25]. At the end of the video session, the CtR group was asked to actively do the breathing exercises and ABCT in the PtR and to imagine along with the instructions in the video recording; commands were given accordingly.

### 2.5. Statistical Analysis

In order to determine the sample size in this study, power analysis was calculated using the G*Power3.1 program, with reference to a similar article [26]. The Cochran formula was used to determine the sample size [27]. In the power analysis performed to obtain the largest sample size, the correlation coefficient was 0.5, the alpha value was accepted as 0.05, and the margin of error was 0.2. It was calculated that at least a total of 22 patients should be included in this study in order to obtain 95% power at a 95% confidence level.

The analysis of the data was carried out using the SPSS 25 package program. A Chi-square test was used for comparisons between two qualitative variables. The Mann–Whitney U test was used for comparisons between two category qualitative variables and quantitative variables, and the Wilcoxon test was used for comparisons between two repeated measurements. Before the comparisons were made in terms of the changes between the groups, difference scores were calculated between the post- and pre-application values. Calculated values were compared with the Mann–Whitney U test. The type I error rate was taken as 0.05 in this study.

We used data mining methods to plot decision trees to extract hidden patterns within patients on whether the treatment would be beneficial. The same methods have been used in the distinguished literature [28,29,30,31,32,33]. R software version 4.4.1 environment was used for statistical computing and graphics.

## 3. Results

### 3.1. Participants

In total, 29 patients with severe COPD were screened for eligibility and 26 were included and randomly assigned to the PtR group (n = 13) or CtR group (n = 13; Figure 1). In total, 23 patients remained included in the analysis—11 in the PtR group and 12 in the CtR group. Baseline demographics were similar between the two groups (*p* > 0.05) (Table 1).

### 3.2. Outcomes

Improvements were found in the 6MWT (*p* < 0.05) in both groups significantly after treatment compared with before treatment (Table 2). Improvements were found in all the peripheral muscle strength measurements, the CAT symptom score, LCADL score, and all SGRQ scores (*p* < 0.05) in both groups significantly after treatment compared with before treatment (Table 2). While no significant improvement was observed in the mMRC score or MMSE score (*p* > 0.05) in the PtR group compared with before and after treatment, a significant improvement was observed in the CtR group (*p* < 0.05) (Table 2). There were no significant improvements in blood lactate levels in both groups after treatment compared with pre-treatment (*p* < 0.05) (Table 2). The comparison of difference scores between groups for primary and secondary outcomes is shown in Table 3.

**Figure 3:**
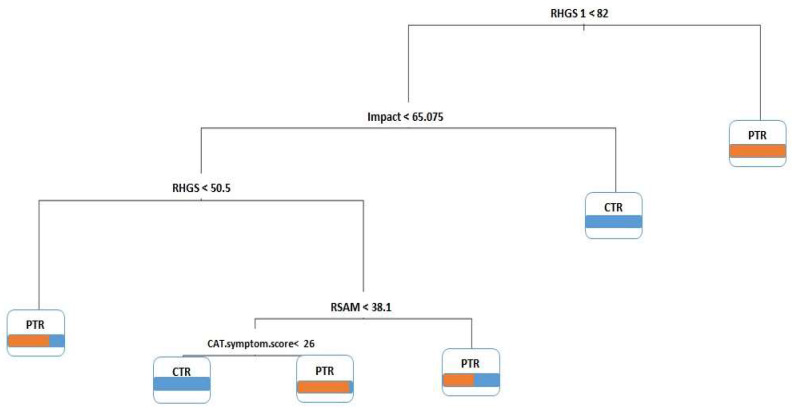
Classification patterns of scores between groups. Meaning of colors: Blue for cognitive telerehabilitation (CTR) group, orange for pulmonary telerehabilitation (PTR) group.

Data mining methods were used to plot decision trees to extract hidden patterns within patients on whether the treatment would be beneficial. Figure 3 demonstrates the classification patterns of scores between groups.

For any test data, rule 1: if (RHGS1 > 82), the participant assigns to the PTR group.

Rule 2: if (RHGS1 < 82) and (impact > 65.075), the participant assigns to the CTR group.

Rule 3: if (RHGS1 < 82) and (impact < 65.075) and (RHGS < 50.5), the participant assigns to the PTR group.

Rule 4: if (RHGS1 < 82) and (impact < 65.075) and (RHGS > 50.5) and (RSAM > 38.1), the participant assigns to the PTR group.

Rule 5: if (RHGS1 < 82) and (impact < 65.075) and (RHGS > 50.5) and (RSAM < 38.1) and (CAT symptom score < 26), the participant assigns to the CTR group.

Rule 6: if (RHGS1 < 82) and (impact < 65.075) and (RHGS > 50.5) and (RSAM < 38.1) and (CAT symptom score > 26), the participant assigns to the PTR group.

After the program was compared with before the average muscle activity was obtained from the superficial EMG measurement, there were statistically significant increases (*p* < 0.05) in the muscle activity of the SCM at rest, inspiratory pressure of 7 cmH_2_O and 14 cmH_2_O, and the muscle activity of the parasternal muscle at 7 cmH_2_O inspiratory pressure in the PtR group. There was a decrease in muscle activity in the SCM and parasternal muscles at 21 cmH_2_O inspiratory pressure (*p* < 0.05) (Table 4). In the CtR group, there was a significant decrease only in SCM muscle activity in the EMG measurement at 14 cmH_2_O inspiratory pressure, and in parasternal muscle activity in the EMG measurement at 21 cmH_2_O inspiratory pressure (*p* < 0.05) (Table 4). Compared with before and after treatment, there was no statistically significant change in the mean muscle activity of the muscles of the other superficial EMG measurement regions in both groups (*p* > 0.05) (Table 4). The comparison of difference scores between groups for EMG measurements is shown in Table 5.

**Figure 4:**
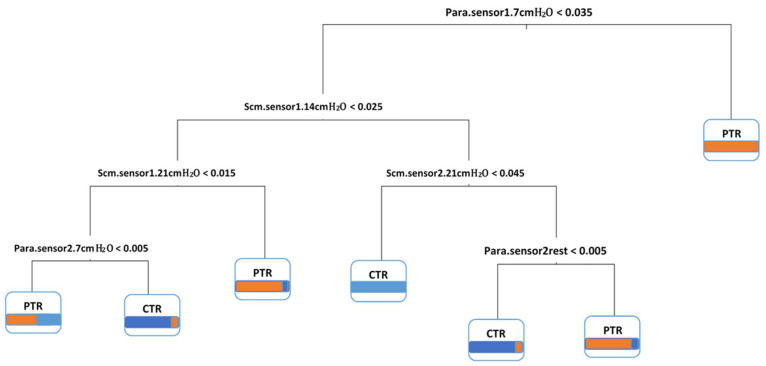
Classification patterns of scores between groups. Meaning of colors: Blue for cognitive telerehabilitation (CTR) group, orange for pulmonary telerehabilitation (PTR) group.

Figure 4 demonstrates the classification patterns for the EMG measurement scores between groups. The extracted rules for decisions in Figure 3 are as follows:

For any test data, rule 1: if (Para-sensor1-7 cmH_2_O > 0.035), the participant assigns to the PTR group.

Rule 2: if (Para-sensor1-7 cmH_2_O < 0.035) and (Scm-sensor1-14 cmH_2_O < 0.025), then (Scm-sensor1-21 cmH_2_O > 0.015), the participant assigns to the PTR group.

Rule 3: if (Para-sensor1-7 cmH_2_O < 0.035) and (Scm-sensor1-14 cmH_2_O < 0.025), then (Scm-sensor1-21 cmH_2_O < 0.015) and (Para-sensor2-7 cmH_2_O > 0.005), the participant assigns to the CTR group.

Rule 4: if (Para-sensor1-7 cmH_2_O < 0.035) and (Scm-sensor1-14 cmH_2_O < 0.025), then (Scm-sensor1-21 cmH_2_O < 0.015) and (Para-sensor2-7 cmH_2_O < 0.005), the participant assigns to the PTR group.

Rule 5: if (Para-sensor1-7 cmH_2_O < 0.035) and (Scm-sensor1-14 cmH_2_O > 0.025), then (Scm-sensor2-21 cmH_2_O < 0.045), the participant assigns to the CTR group.

Rule 6: if (Para-sensor1-7 cmH_2_O < 0.035) and (Scm-sensor1-14 cmH_2_O > 0.025), then (Scm-sensor2-21 cmH_2_O > 0.045) and (Para-sensor2rest < 0. 005), the participant assigns to the CTR group.

Rule 7: if (Para-sensor1-7 cmH_2_O < 0.035) and (Scm-sensor1-14 cmH_2_O > 0.025), then (Scm-sensor2-21 cmH_2_O > 0.045) and (Para-sensor2rest > 0.005), the participant assigns to the PTR group.

## 4. Discussion

We hypothesized the effects of remote pulmonary rehabilitation and motor imaging (MI) and action observation (AO) methods on functional capacity, peripheral muscle strength, respiratory muscle activity, activities of daily living, and quality of life in patients with severe COPD. To the best of our knowledge, our study is the first single-center randomized controlled trial in which CPR using MI and AO methods was performed in respiratory patients and compared with comprehensive synchronous PTR. In our study, 29 patients with CAOH were evaluated and 26 patients who met the criteria were randomized into 2 groups. At the end of 8 weeks, 11 patients for the PtR group and 12 patients for the CtR group were analyzed. According to the data obtained from our study, it was determined that both rehabilitation methods provided improvement in physical and functional parameters and there was no difference between them. Especially in the CtR group, we found that motor imagery and action observation methods were potential methods that could provide alternative support to elderly respiratory patients who have difficulty in exercising.

Godtfredsen et al., in their study in 2020 in which they compared standard PR and PtR applied for ten weeks in severe COPD, found that, while a significant improvement was found in the result of 6MWT after the intervention and at the third-month follow-up, this improvement could not be maintained in both groups at the twelve-month follow-up [7]. Park et al., in 2017 in their study that lasted four weeks (three days a week on twenty-five patients with chronic stroke who were hospitalized), divided the patients into the control group (where only the landscape video was watched) and the AO method group (which included the ambulation training). As a result of their studies, significant improvement was achieved in both groups in the ten-meter walking test (10MWT), while walking function and ambulation confidence improved significantly in the experimental group compared with the control group after the program [34]. In our study, PtR and CtR were compared for the first time in COPD patients and it was found that both significantly increased the result of 6MWT, which is an indicator of functional capacity. The significant improvement in the 6MWT results with CtR may be that we had active walking in our program and that the walking function, which was imagined every night before going to bed, led to neuronal activation in the relevant brain regions. In our study, it is not known whether the effects of the methods were preserved or not, since long-term follow-up was not performed after the end of the program. Hansen et al., in their study in 2020 that compared the effectiveness of supervised PRT and conventional PR in severe COPD patients, found that there was no difference between the groups in the results of 6MWT and thirty-second sit and stand test, and that the methods were not superior to each other and had a positive effect on the results [20]. Parallel to our study, it was observed in their studies that PtR significantly improved the CAT symptom score. In their study in 2016, Tsai et al. reported that synchronous videoconference-based supervised TR clinically increased 6MWT and other gait test performances, and, unlike our study, they did not report significant CAT symptom scores compared with the control group, who did not receive exercise intervention after rehabilitation. While Tsai applied aerobic walking and strengthening exercises, in our study, ACBT, which is an extra-comprehensive breathing exercises and secretion beat technique, was actively applied to both groups.

To the best of our knowledge, there is no study in the literature examining the effect of pulmonary and cognitive rehabilitation on blood lactate levels. In our study, although there was a decrease in blood lactate levels after rehabilitation in both groups, no statistically significant improvement occurred. The blood lactate level we looked at here was not the acute lactate level after planned exercise with a certain exercise intensity as in lactate studies in the literature, but the blood lactate accumulation level of patients between 8 weeks before and 8 weeks after an 8-week rehabilitation program in general. Here, we aimed to investigate how rehabilitation after 8 weeks affected the existing lactate metabolism of a routine COPD patient and whether it provided improvement. It is known that, even in mild and moderate COPD patients, the oxidative capacity is damaged and the blood lactate level increases during intense activity [35]. Normally, in such a situation, we can understand that the blood lactate metabolism of a COPD patient who has not undergone any rehabilitation program is distressed. We hypothesized that an 8-week rehabilitation program including exercises such as breathing and walking would improve the blood lactate metabolism of these patients. The reasons for this may be that our study was conducted on severe COPD patients whose oxidative capacities were more severely impaired, and the eight-week rehabilitation period was insufficient to provide a significant enough improvement in oxidative capacity.

It is mentioned in the literature that MI and AO methods can affect muscle strength [36,37,38]. On the other hand, our study is the first study in which PR was applied with CtR methods and its effect was examined. In our study, there was an increase in muscle strength in both the PtR and CtR groups, without either being superior. While it is expected that muscle strength increase will occur with PR, muscle strength increase occurred in our study by applying the MI + AO method without active muscle strengthening exercises. The literature also states that people with COPD have deficits in muscle strength and functional status impairments, which increases their risk of developing frailty. Frail COPD patients also have higher mortality than non-frail patients and are more likely to have sarcopenia, increased morbidity, and more severe disease [39]. As a contribution to the literature, our study provides the combined use of MI and AO applications in respiratory patients in order to maintain muscle strength in cases where exercise is not possible or sufficient. There is a need for further research in the field of pulmonary diseases using the MI and AO methods of PR practices due to insufficient studies in the literature.

Franklin et al., in 2019 in their study in which the AO application for the thumb evaluated the change in cortical activity and muscle activity with the transcranial magnetic stimulation method and EMG measurement, experienced that significantly increased motor-evoked potential amplitudes occurred in the abductor pollicis brevis during AO [10]. Ours is the first study to compare respiratory muscle activity in PtR and CtR methods. In our study, the increase in SCM and parasternal muscle activity in the PtR group, especially at rest and under low pressure, but the absence of CtR may be due to the fact that the PtR group actively worked on peripheral muscle strengthening and the activity of these muscles increased with the force diffusion principle. A possible reason for the decrease in SCM and parasternal muscle activity in both groups at high pressures may be the occurrence of contraction with reduced muscle activity under forced pressure without the need for unnecessary muscle activity. There may be no significant changes in the activity of the diaphragm, the diaphragm being deep and our EMG measurement sensors detecting the superficial changes and not detecting the diaphragm sufficiently.

In the literature, while EMG evaluation was carried out in studies during MI or AO methods, we applied an eight-week training and measured inspiratory pressure and resting independent of the methods. In the literature, similar to our EMG usage method, only Weiliang et al.’s study examined the superficial muscle activity of the SCM, parasternal, and diaphragm muscles of COPD patients during treadmill exercises [22]. While in the study, muscle activity was examined during exercise intensity increases, in our study, eight-week rehabilitation programs were applied and examined.

As a result of our study, although neither group was superior to each other in our measurement parameters, it was seen that the CtR method, which was applied in addition to PtR methods but did not include active muscle strengthening, had a more comprehensive benefit.

This study has some limitations. Initially, in our study, both groups were at risk in terms of severe COPD patients, although no adverse events occurred, since instantaneous vital signs were not followed up by a concurrent physiotherapist. This risk was reduced by good patient education and by checking vital signs regularly during the exercise by the physiotherapist for the PtR group. Another limitation was that it was not clear whether the surface EMG sensor used adequately measured the diaphragm activity (which is a deep muscle). Another limitation was that we could not observe whether the patients applied the program exactly as we wanted, since the CtR group was executed based on asynchronous video commands. Another limitation of this study was that we could not perform long-term follow-up of the patients and therefore we could not emphasize the quality of life parameters in the results or the discussion section.

## 5. Conclusions

In conclusion, our study showed that the CtR method, which includes AO and MI techniques, is effective in patients with severe COPD, unlike the PtR method. Cognitive telerehabilitation can be a powerful alternative rehabilitation method in severe respiratory patients who cannot tolerate active exercise programs and/or have problems with transfer to the hospital.

## Figures and Tables

**Table 1:** Baseline characteristics of patients.

	PTR (N = 11)	CTR (N = 12)	U	*p* Value ^a^
Demographic characteristics				
Gender (male/female), n (%)	9/2 (81.8/18.2)	11/1 (91.7/8.3)	0.007 ^b^	0.936 ^b^
Age (year)	65 (54–79)	62 (49–79)	51	0.379
Anthropometry				
BMI (kg/m^2^)	28.13 (17.36–33.36)	24.535 (18.93–33.22)	42	0.151
Smoking				
Yes (%)	8 (72.7)	9 (75)	0.015 ^b^	0.901 ^b^
No (%)	3 (27.3)	3 (25)		
Lung function *				
FVC measured (L)	1.95 (1.13–2.97)	1.865 (0.38–2.63)	53.5	0.449
FVC (%)	59 (29–76)	47 (16–62)	37	0.079
FEV1 measured (L)	0.96 (0.55–1.85)	0.76 (0.25–1.22)	50	0.347
FEV1(%)	34 (15–60)	26 (13–38)	43	0.169
FEV1/FVC measured	48 (28–72)	50.5 (30–77)	62	0.833

PTR—pulmonary telerehabilitation group. CTR—cognitive telerehabilitation group. BMI—body mass index. FVC—functional vital capacity. FEV1—forced expiratory volume first second volume. FEV1/FVC—Tiffeneau index. Data are n (%) or min–max (min–max). ^a^ Mann–Whitney U-test. ^b^ Chi-square test; significance level *p* < 0.05. * Lung functions were determined by spirometric pulmonary function test at the hospital.

**Table 2:** Primary and secondary outcomes before and after treatment.

	PTR (N = 11)				CTR (N = 12)			
	Before	After	Z	*p* ^a^	Before	After	Z	*p* ^a^
6MWT (min)	330 (132–528)	572 (297–748)	−2.941	0.003 ^a^	346.5 (176–616)	517 (374–748)	−3.066	0.002 ^a^
Muscle strength (Lbs)								
RSFM	29.2 (16.1–41.5)	46.7 (22.4–60.5)	−2.936	0.003 ^a^	27.5 (13.2–35.3)	42.8 (14–55)	−3.059	0.002 ^a^
LSFM	27.2 (19.3–44.1)	44 (22.6–55.5)	−2.934	0.003 ^a^	26.4 (13.2–39.8)	38.4 (25–52.8)	−3.061	0.002 ^a^
RSAM	25.3 (16.3–38)	42.5 (21.3–49.7)	−2.934	0.003 ^a^	22.2 (9.2–31.8)	33.35 (16.9–43.5)	−3.059	0.002 ^a^
LSAM	27.4 (15.6–36)	34.2 (19.9–54)	−2.934	0.003 ^a^	23.35 (14.6–30)	34.25 (23.2–41.5)	−3.061	0.002 ^a^
REFM	30.2 (18.9–46.6)	45.6 (27.2–61)	−2.934	0.003 ^a^	31.05 (0–39.7)	42.6 (0–51.7)	−2.578	0.010 ^a^
LEFM	30.6 (17.9–54.9)	44 (26.2–61)	−2.934	0.003 ^a^	26.5 (18.4–49.9)	38.95 (31.4–55.4)	−2.473	0.013
REEM	28.5 (16.9–40.7)	37.1 (18.2–46.1)	−2.934	0.003 ^a^	24 (10.9–39.3)	35.1 (20–45.6)	−3.059	0.002 ^a^
REEM	26.9 (15.4–34.6)	33 (19.1–45.2)	−2.845	0.004 ^a^	23.75 (11.7–35.6)	30.15 (24.2–38.5)	−2.276	0.023 ^a^
RHFM	31 (19.9–41.7)	50 (35.9–68.4)	−2.934	0.003 ^a^	30.4 (14.1–41.5)	47.25 (27.7–62.2)	−3.061	0.002 ^a^
LHFM	28.2 (21.2–50.1)	45.2 (28.9–62.1)	−2.934	0.003 ^a^	28.95 (17.3–38.4)	41.95 (17.3–51.5)	−2.934	0.003 ^a^
RKEM	16.9 (11.8–32.4)	28.3 (20–43.3)	−2.934	0.003 ^a^	20.2 (9.7–26.7)	27 (12.6–43.7)	−3.059	0.002 ^a^
LKEM	17.4 (12.4–34.2)	24.6 (19.6–76.3)	−2.934	0.003 ^a^	18.2 (7.6–26)	28.35 (11.1–34.7)	−3.059	0.002 ^a^
RDFM	29.3 (18.7–36.7)	47.9 (34.4–76.3)	−2.934	0.003 ^a^	30.75 (19.5–40)	48.3 (35–54.6)	−3.061	0.002 ^a^
LDFM	29.9 (19.4–41.9)	47.6 (35.8–71.9)	−2.803	0.005 ^a^	32.45 (15.3–42)	45.8 (35.7–55.6)	−3.059	0.002 ^a^
RHGS	45 (35–91)	75 (38–96)	−2.194	0.028 ^a^	49 (40–84)	64.5 (42–91)	−2.807	0.005 ^a^
RHGS	51 (26–88)	69 (32–101)	−2.606	0.009 ^a^	47 (19–75)	60 (35–81)	−2.805	0.005
CAT symptom score	30 (7–37)	22 (1–33)	−2.67	0.008 ^a^	30 (24–38)	20 (4–36)	−2.805	0.005
mMRC score	3 (1–4)	3 (1–4)	−1.633	0.102	4 (3–4)	3.5 (2–4)	−2.236	0.025 ^a^
Blood lactate level	8 (2.1–10.5)	4.4 (1.9–8)	−1.66	0.097	4.95 (1–12.1)	4.1 (1–8.6)	−1.58	0.114
LCADL score	29 (23–62)	17 (10–38)	−2.936	0.003 ^a^	29.5 (22–41)	19 (11–43)	−2.763	0.006 ^a^
SGRQ scores								
Symptom	79.65 (25.7–92.96)	39.28 (5.27–72.78)	−2.934	0.003 ^a^	79.205 (39.13–98.01)	57.525 (0–78.91)	−3.059	0.002 ^a^
Activity	85.87 (60.38–100)	54.5 (23.3–85.81)	−2.934	0.003 ^a^	96.685 (67.26–100)	70.235 (29.23–100)	−2.521	0.012 ^a^
Impact	46.08 (17–82.46)	32.67 (11.04–44.09)	−2.845	0.004 ^a^	71.065 (5.88–94.97)	39.64 (9.2–91.29)	−2.845	0.004 ^a^
Total	61.42 (39.63–89.36)	45.21 (18.72–56.25)	−2.934	0.003 ^a^	82.61 (38.04–95.11)	52.665 (19.31–89.64)	−3.059	0.002 ^a^
MMSE score	29 (24–30)	30 (24–30)	−1.826	0.068	25 (24–30)	30 (24–30)	−2.682	0.007 ^a^

PTR—pulmonary telerehabilitation group. CTR—cognitive telerehabilitation group. 6MWT—six-minute walk test. Min—minute. RSFMStrength—right shoulder flexor muscle strength. LSFMStrength—left shoulder flexor muscle strength. RSAMStrength—right shoulder abductor muscle strength. LSAMStrength—left shoulder abductor muscle strength. REFMStrength—right elbow flexor muscle strength. LEFMStrength—left elbow flexor muscle strength. REEMStrength—right elbow extensor muscle strength. REEMStrength—left elbow extensor muscle strength. RHFMStrength—right hip flexor muscle strength. LHFMStrength—left hip flexor muscle strength. RKEMStrength—right knee extensor muscle strength. LKEMStrength—left knee extensor muscle strength. RDFMStrength—right dorsi flexor muscle strength. LDFMStrength—left dorsi flexor muscle strength. RHGS—right hand grip strength. RHGS—left hand grip strength. mMRC—modified medical research council dyspnea scale. LCADL—London chest activity of daily living scale. SGRQ—St. George’s Respiratory Questionnaire. MMSE—mini mental state examination. Data are median value (min–max). ^a^ Wilcoxon test; significance level *p* < 0.05.

**Table 3:** Comparison of difference scores between groups for primary and secondary outcomes.

	PTR (N = 11)	CTR (N = 12)		
	Delta (∆)	Delta (∆)	U	*p* ^b^
6MWT (min)	220 (66–396)	154 (66–374)	37.5	0.079
Muscle strength (Lbs)				
RSFM	15.6 (1.6–22.7)	13.6 (0.8–25.3)	64	0.928
LSFM	11.4 (1–26.6)	11.4 (5.7–29.8)	64.5	0.928
RSAM	13.6 (1.9–21.1)	9.65 (3.4–20.4)	57	0.608
LSAM	11.4 (0.4–18)	12.55 (1.8–15.9)	55	0.525
REFM	9.6 (2.3–26.7)	9.25 (−7.3–31.1)	64	0.928
LEFM	9.2 (3.3–26.3)	11.9 (−17.5–24.4)	53	0.449
REEM	7.1 (0.2–12.7)	10.2 (0.6–18.7)	37	0.079
REEM	5.7 (−0.3–13.9)	7.4 (−11.4–15.5)	49	0.316
RHFM	18.7 (9.3–34.5)	14.85 (9.9–32.3)	47.5	0.26
LHFM	13.8 (7.7–23.9)	12.8 (0–22.3)	47	0.26
RKEM	10.1 (3.1–21)	10.45 (0.3–25.9)	56	0.566
LKEM	7.6 (4.1–58.9)	11.55 (0.1–18.2)	62	0.833
RDFM	17 (6.8–39.6)	16.75 (3.6–25.3)	59.5	0.695
LDFM	20.2 (0–39.8)	13.25 (7.1–25.4)	57.5	0.608
RHGS	5 (−5–45)	12 (0–24)	64	0.928
RHGS	8 (−2–57)	10 (0–32)	61	0.786
CAT symptom score	−7 (−19–0)	−10 (−23–0)	62.5	0.833
mMRC score	0 (−2–0)	0 (−1–0)	59	0.695
Blood lactate level	−1 (−8.6–2)	−1.65 (−6–6.2)	60	0.74
LCADL score	−11 (−42–4)	−9.5 (−22–5)	51	0.379
SGRQ scores				
Symptom	−30.84 (−79.77–5.2)	−20.04 (−51.31–13.15)	61	0.786
Activity	−32.19 (−58.27–6.84)	−23.1 (−38.09–0)	43	0.169
Impact	−19.13 (−43.65–1.01)	−22.76 (−51.14–3.32)	58	0.651
Total	−23.86 (−51.63–9.09)	−21.34 (−45.63–2.46)	57.5	0.608
MMSE score	0 (0–5)	3 (0–5)	36	0.069

PTR—pulmonary telerehabilitation group. CTR—cognitive telerehabilitation group. 6MWT—six-minute walk test. min—minute. RSFMStrength—right shoulder flexor muscle strength. LSFMStrength—left shoulder flexor muscle strength. RSAMStrength—right shoulder abductor muscle strength. LSAMStrength—left shoulder abductor muscle strength. REFMStrength—right elbow flexor muscle strength. LEFMStrength—left elbow flexor muscle strength. REEMStrength—right elbow extensor muscle strength. REEMStrength—left elbow extensor muscle strength. RHFMStrength—right hip flexor muscle strength. LHFMStrength—left hip flexor muscle strength. RKEMStrength—right knee extensor muscle strength. LKEMStrength—left knee extensor muscle strength. RDFMStrength—right dorsi flexor muscle strength. LDFMStrength—left dorsi flexor muscle strength. RHGS—right hand grip strength. RHGS—left hand grip strength. mMRC—modified medical research council dyspnea scale. LCADL—London chest activity of daily living scale. SGRQ —St. George’s Respiratory Questionnaire. MMSE—mini mental state examination. Data are median value (min–max). ^b^ Mann–Whitney U-test; significance level *p* < 0.05.

**Table 4:** Results of superficial EMG measurement before and after treatment.

	PTR (N = 11)				CTR (N = 12)			
	Before	After	Z	*p* ^a^	Before	After	Z	*p* ^a^
EMG (millivolts)								
Scm-sensor1rest	0.02 (0–4.97)	0 (0–0.48)	−2.023	0.043	0 (0–0.29)	0 (0–0.7)	−0.271	0.786
Scm-sensor2rest	0.09 (0–4.32)	0.01 (0–0.11)	−2.134	0.033	0.015 (0–0.1)	0.005 (0–4.59)	−0.677	0.498
Para-sensor1rest	0.01 (0–2.74)	0.01 (0–0.03)	−1.549	0.121	0 (0–0.03)	0.01 (0–0.56)	−2.259	0.887
Para-sensor2rest	0.01 (0–2.52)	0.01 (0–0.1)	−0.841	0.400	0 (0–0.17)	0.01 (0–1.3)	−1.025	0.722
Di-sensor1rest	0 (0–0.28)	0 (0–0.12)	−0.086	0.931	0 (0–4.78)	0.01 (0–0.08)	−0.359	0.720
Di-sensor2rest	0 (0–0.1)	0 (0–0.91)	−0.512	0.609	0.01 (0–5.57)	0.01 (0–0.07)	−1.189	0.234
Scm-sensor1-7 cmH_2_O	0.04 (0.01–2.88)	0.02 (0–1.08)	−2.106	0.035	0.03 (0–0.69)	0.01 (0–2.89)	−0.868	0.386
Scm-sensor2-7 cmH_2_O	0.14 (0.02–5.84)	0.04 (0–0.91)	−1.29	0.197	0.035 (0–0.2)	0.01 (0–0.87)	−0.079	0.937
Para-sensor1-7 cmH_2_O	0.02 (0.01–2.96)	0.01 (0–0.03)	−2.252	0.024	0.01 (0–0.01)	0.01 (0–0.03)	−0.962	0.336
Para-sensor2-7 cmH_2_O	0.01 (0–2.52)	0.01 (0–0.1)	−0.841	0.400	0.01 (0–0.03)	0.01 (0–0.23)	−1.611	0.107
Di-sensor1-7 cmH_2_O	0.02 (0–2.81)	0.01 (0–0.35)	−0.631	0.528	0.01 (0–1.94)	0.015 (0–0.83)	−0.103	0.918
Di-sensor2-7 cmH_2_O	0.01 (0–2.59)	0.01 (0–0.13)	−1.609	0.108	0.01 (0–0.19)	0.01 (0–0.35)	−0.341	0.733
Scm-sensor1-14 cmH_2_O	0.05 (0.01–3.09)	0.01 (0–0.03)	−2.677	0.007	0.03 (0–0.37)	0.03 (0.01–0.1)	−0.36	0.719
Scm-sensor2-14 cmH_2_O	0.18 (0.06–2.63)	0.03 (0–0.52)	−2.224	0.026	0.03 (0–0.12)	0.095 (0.01–3.31)	−2.315	0.021
Para-sensor1-14 cmH_2_O	0.01 (0–2.95)	0.01 (0–0.03)	−0.986	0.324	0.01 (0–0.03)	0.015 (0–0.3)	−1.437	0.151
Para-sensor2-14 cmH_2_O	0.01 (0–9.24)	0.01 (0–0.02)	−1.897	0.058	0 (0–0.11)	0.01 (0–0.06)	−1.63	0.103
Di-sensor1-14 cmH_2_O	0.03 (0–2.93)	0.01 (0–0.05)	−1.494	0.135	0.01 (0–0.13)	0.01 (0.01–0.27)	−1.251	0.211
Di-sensor2-14 cmH_2_O	0.01 (0–2.52)	0.01 (0–0.06)	−1.542	0.123	0.01 (0–0.62)	0.01 (0–0.1)	−0.314	0.753
Scm-sensor1-21 cmH_2_O	0.17 (0.02–2.93)	0.02 (0–0.08)	−2.504	0.012	0.035 (0–0.07)	0.015 (0–0.3)	−0.46	0.646
Scm-sensor2-21 cmH_2_O	0.06 (0–2.7)	0.02 (0–0.54)	−1.379	0.168	0.03 (0.01–0.25)	0.035 (0–2.73)	−1.245	0.213
Para-sensor1-21 cmH_2_O	0.01 (0.01–2.96)	0.01 (0–0.03)	−2.226	0.026	0.01 (0–0.01)	0.01 (0–0.07)	−1.983	0.047
Para-sensor2-21 cmH_2_O	0.01 (0–2.58)	0.01 (0–0.02)	−1.843	0.065	0.01 (0–1.25)	0.01 (0–0.12)	−0.17	0.865
Di-sensor1-21 cmH_2_O	0.04 (0–2.93)	0.01 (0.01–0.48)	−1.719	0.086	0.01 (0–0.05)	0.03 (0–0.38)	−1.701	0.089
Di-sensor2-21 cmH_2_O	0.01 (0–2.82)	0.01 (0–0.06)	−1.219	0.223	0.01 (0–0.63)	0.02 (0–0.45)	0	1.000

Average data obtained from the application of the EMG logger in millivolts of physiological activities in the motor unit during muscle contraction. PTR—pulmonary telerehabilitation group. CTR—cognitive telerehabilitation group. SCM—electromyographic value of sternocleidomastoid muscle. Para—electromyographic value of parasternal muscle. Di—electromyographic value of diaphragm muscle. Data are median value (min–max). ^a^ Wilcoxon test. Significance level *p* < 0.05.

**Table 5:** Comparison of difference scores between groups for EMG measurements drawn.

	PTR (N = 11)	CTR (N = 12)		
	Delta (∆)	Delta (∆)	U	*p* ^b^
EMG (millivolts)				
Scm-sensor1rest	0 (−4.97–0)	0 (−0.29–0.7)	41.000	0.134
Scm-sensor2rest	−0.08 (−4.32–0.05)	0 (−0.07–4.5)	35.000	0.059
Para-sensor1rest	0 (−2.72–0.01)	0.01 (−0.02–0.56)	20.000	0.004
Para-sensor2rest	0 (−2.51–0.08)	0.005 (−0.17–1.3)	44.000	0.190
Di-sensor1rest	0 (−0.28–0.12)	0.005 (−4.78–0.07)	58.000	0.651
Di-sensor2rest	0 (−0.1–0.91)	−0.01 (−5.57–0.07)	60.000	0.740
Scm-sensor1-7 cmH_2_O	−0.01 (−2.88–0.02)	−0.01 (−0.66–2.86)	52.000	0.413
Scm-sensor2-7 cmH_2_O	−0.06 (−5.84–0.87)	−0.01 (−0.2–0.84)	47.000	0.260
Para-sensor1-7 cmH_2_O	−0.01 (−2.95–0.01)	0 (−0.01–0.03)	28.000	0.019
Para-sensor2-7 cmH_2_O	−0.01 (−2.59–0.1)	0.01 (−0.03–0.22)	39.000	0.104
Di-sensor1-7 cmH_2_O	0 (−2.81–0.35)	0.005 (−1.93–0.82)	58.500	0.651
Di-sensor2-7 cmH_2_O	0 (−2.59–0.03)	0 (−0.19–0.33)	45.500	0.211
Scm-sensor1-14 cmH_2_O	−0.03 (−3.07–0)	0.005 (−0.31–0.07)	24.500	0.009
Scm-sensor2-14 cmH_2_O	−0.17 (−2.62–0.46)	0.08 (−0.06–3.19)	11.000	<0.001
Para-sensor1-14 cmH_2_O	0 (−2.95–0.02)	0.005 (−0.02–0.3)	45.000	0.211
Para-sensor2-14 cmH_2_O	0 (−9.24–0.01)	0.01 (−0.11–0.05)	22.000	0.006
Di-sensor1-14 cmH_2_O	0 (−2.92–0.01)	0.005 (−0.04–0.14)	36.000	0.069
Di-sensor2-14 cmH_2_O	0 (−2.51–0.05)	0 (−0.6–0.1)	50.000	0.347
Scm-sensor1-21 cmH_2_O	−0.14 (−2.92–0.02)	0 (−0.05–0.27)	28.000	0.019
Scm-sensor2-21 cmH_2_O	−0.05 (−2.68–0.48)	0.015 (−0.19–2.48)	35.500	0.059
Para-sensor1-21 cmH_2_O	−0.01 (−2.96–0)	0.005 (−0.01–0.06)	19.000	0.003
Para-sensor2-21 cmH_2_O	−0.01 (−2.57–0.01)	0 (−1.24–0.12)	42.500	0.151
Di-sensor1-21 cmH_2_O	−0.03 (−2.89–0.07)	0.015 (−0.05–0.35)	31.500	0.032
Di-sensor2-21 cmH_2_O	0 (−2.82–0.01)	0 (−0.63–0.06)	52.000	0.413

Average data obtained from the application of the EMG logger in millivolts of physiological activities in the motor unit during muscle contraction. PTR—pulmonary telerehabilitation group. CTR—cognitive telerehabilitation group. SCM—electromyographic value of sternocleidomastoid muscle. Para—electromyographic value of parasternal muscle. Di—electromyographic value of diaphragm muscle. Data are median value (min–max). ^b^ Mann–Whitney U-test. Significance level *p* < 0.05.

## Data Availability

The dataset originated from the trial is available upon request to the corresponding author.
